# Conferring extracellular matrix affinity enhances local therapeutic efficacy of anti-TNF-α antibody in a murine model of rheumatoid arthritis

**DOI:** 10.1186/s13075-019-2075-8

**Published:** 2019-12-23

**Authors:** Kiyomitsu Katsumata, Jun Ishihara, Kazuto Fukunaga, Ako Ishihara, Eiji Yuba, Erica Budina, Jeffrey A. Hubbell

**Affiliations:** 1 Pritzker School of Molecular Engineering, University of Chicago, Chicago, IL 60637 USA; 2 Present Address: Astellas Pharma Inc., Tsukuba, Ibaraki 305-8585 Japan; 3 Present Address: FUJIFILM Corporation, Kanagawa, 258-8577 Japan; 4 Department of Applied Chemistry, Osaka Prefecture University, Osaka, 599-8531 Japan; 5 Committee on Immunology, University of Chicago, Chicago, IL 60637 USA

**Keywords:** Extracellular matrix binding peptide, PlGF-2_123-144_ peptide, Bioengineering, Joint targeting, Localized therapy, Collagen antibody-induced arthritis model

## Abstract

**Background:**

Although disease in a majority of rheumatoid arthritis (RA) patients is often initially limited to one or a few joints, currently approved medications including anti-tumor necrosis factor-α antibody (α-TNF) are injected systemically. Given that α-TNF systemic injection typically does not cure RA and involves risk of treatment-related adverse events, one possible approach to enhance therapeutic efficacy and reduce α-TNF systemic exposure is to retain the antibodies in arthritic joints after local administration. The aim of this study was to evaluate the approach of conferring extracellular matrix (ECM) binding affinity to α-TNF antibodies in a RA model.

**Methods:**

α-TNF was chemically conjugated with a promiscuous ECM-binding peptide derived from placenta growth factor 2 (PlGF-2_123-144_). The binding activity of PlGF-2_123-144_-conjugated α-TNF (PlGF-2_123-144_-α-TNF) against ECM proteins was assessed by ELISA and by immunostaining on human cartilage specimens. The effect of conjugation on antibody function was assessed as a neutralizing activity against osteoclast differentiation. Retention at the injection site and therapeutic efficacy of PlGF-2_123-144_-α-TNF were tested in a collagen antibody-induced arthritis (CAIA) model in the mouse.

**Results:**

PlGF-2_123-144_ peptide conjugation conferred α-TNF with affinity to ECM proteins without impairment of antigen recognition. PlGF-2_123-144_-α-TNF locally injected at a paw in the CAIA model was retained for at least 96 h at the injection site, whereas unmodified α-TNF was dispersed rapidly after injection. Local treatment with unmodified α-TNF did not suppress the arthritis score relative to isotype controls. By contrast, local administration of PlGF-2_123-144_-α-TNF suppressed arthritis development almost completely in the treated paw even at a 1000× lower dose.

**Conclusion:**

These data demonstrate that retention of α-TNF in arthritic joints can suppress arthritis development and enhance therapeutic efficacy. This simple bioengineering approach of ECM-binding peptide conjugation offers a powerful and clinically translational approach to treat RA.

## Background

Rheumatoid arthritis (RA) is a chronic, progressive autoimmune disease characterized by the formation of hyperplastic synovial pannus tissue, which causes joint destruction [1, 2]. Biological therapies to block cytokine signals in the human body, such as anti-TNFα (α-TNF) and anti-IL-6 receptor antibodies, are powerful approaches for treating RA [3–6]. However, currently approved medications show incomplete response in terms of joint damage by persistent synovitis even in patients in clinical remission, and they have the possibility of significant side effects by suppressing systemic immunity [7–11]. Arthritis is classified as monoarthritis, oligoarthritis, and polyarthritis, by the numbers of joints in which symptoms appear. Many polyarthritic disorders initially present as a monoarthritis. In patients with recent-onset arthritis, monoarticular involvement (38.3%) is more common than oligoarticular (34.1%) or polyarticular (24.6%) disease [12]. Considering the worse prognosis of polyarticular arthritis, early intervention is critical to maximize outcomes [12, 13].

Direct intra-articular administration of drugs is a reasonable approach to enhance their local efficacy and reduce systemic adverse effects and is relevant for the treatment of monoarthritis and oligoarthritis. However, drugs injected into the joint are rapidly cleared via lymphatics or sub-synovial capillaries in the absence of a retention strategy [14]. In addition, the clearance rate of macromolecules from inflamed joints is increased in patients with RA due to enhanced drainage from the joint space by greater synovial lymph flow [15]. Although intra-articular α-TNF injections have been shown to be well tolerated and improve clinical signs in RA patients in small clinical trials, the therapeutic effect was limited and transient [16, 17]. These results indicate that intra-articular injection is a promising approach for effective therapy, but requires an approach for local tissue retention.

Heparin-binding domains (HBDs) derived from growth factors (GFs) bind to HBDs derived from a variety of extracellular matrix (ECM) proteins [18–22]. By screening the binding of GFs to a variety of ECM proteins, we have discovered that the HBD of placenta growth factor-2 (PlGF-2_123–144_) has an exceptionally high affinity for multiple ECM proteins [23]. Chemical conjugation of the PlGF-2_123–144_ peptide to immune checkpoint blockade and immune agonist antibodies enhanced antibody retention within tumors when injected near the tumor, improving therapeutic efficacy and safety by limiting systemic exposure [24, 25].

Here, we have hypothesized that utilizing ECM-binding technology for prolonged tissue retention would improve the ability of an anti-inflammatory cytokine antibody to effectively suppress RA development. We engineered α-TNF conjugated with PlGF-2_123–144_ peptide for RA therapy. We tested whether the enhanced retention of α-TNF in tissue surrounding the joints would improve its local efficacy against arthritis development.

## Methods

### Synthesis of peptide-conjugated antibody

The synthesis of PlGF-2_123-144_-α-TNF was performed as described previously [24]. Rat anti-mouse TNFα antibody (clone XT3.11, Bio X Cell, West Lebanon, NH, USA; or clone MP6-XT22, BioLegend, San Diego, CA, USA) was incubated with an excess amount of sulfo-SMCC or SM (PEG)_4_ crosslinker (Thermo Fisher Scientific, Waltham, MA, USA) for 30 min at room temperature. Unreacted crosslinker was removed using a Zeba spin desalting column (Thermo Fisher Scientific), and then 15-fold molar excess of PlGF-2_123-144_ peptide (RRRPKGRGKRRREKQRPTDCHL) was added and reacted for 1 h at room temperature for conjugation to the thiol moiety on the C residue. The peptide had been synthesized with > 95% purity by Genscript (Piscataway, NJ, USA).

### Sodium dodecyl sulfate-polyacrylamide gel electrophoresis

Sodium dodecyl sulfate-polyacrylamide gel electrophoresis (SDS-PAGE) was performed on 4–20% gradient gels (Bio-Rad, Hercule, CA, USA) after α-TNF was reduced with 10 mM DTT. After electrophoresis, gels were stained with SimplyBlue SafeStain (Thermo Fisher Scientific) according to the manufacturer’s instruction. Gel images were acquired with the ChemiDoc XRSþ system (Bio-Rad).

### Matrix-assisted laser desorption/ionization–time-of-flight mass spectrometry

Antibodies were analyzed by Matrix-assisted laser desorption/ionization–time-of-flight mass spectrometry (MALDI-TOF MS) using a Bruker Ultraflextreme MALDI TOF/TOF instrument. All spectra were collected with acquisition software Bruker flexControl™ and processed with analysis software Bruker flexAnalysis™. First, a saturated solution of the matrix, α-cyano-4-hydroxycinnamic acid (MilliporeSigma, Billerica, MA, USA), was prepared in 50:50 (v/v) acetonitrile:(1% trifluoroacetic acid in water) as a solvent. The analyte in phosphate-buffered saline (PBS; 5 μL, 0.1 mg/mL) and the matrix solution (25 μL) were then mixed, and 1 μL of that mixture was deposited on the MTP 384 ground steel target plate. The drop was allowed to dry in a nitrogen gas flow, which resulted in the formation of uniform sample/matrix co-precipitate. All samples were analyzed using the high mass linear positive mode method with 5000 laser shots at a laser intensity of 75%. The measurements were externally calibrated at three points with a mix of carbonic anhydrase, phosphorylase B, and bovine serum albumin.

### Enzyme-linked immunosorbent assay

Ninety-six well Enzyme-linked immunosorbent assay (ELISA) plates (Greiner Bio-One, Monroe, NC, USA) were coated with recombinant human ECM proteins (10 μg/mL): fibronectin (MilliporeSigma), decorin (Abcam, Cambridge, MA, USA), collagen I (MilliporeSigma), collagen II (MilliporeSigma), collagen III (MilliporeSigma), or collagen IV (MilliporeSigma); or 1 μg/mL of recombinant murine TNFα (PeproTech, Princeton, NJ, USA) in PBS overnight at 37 °C, followed by blocking with 2% BSA in PBS with 0.05% Tween 20 (PBS-T) for 1 h at room temperature. Then, wells were washed with PBS-T and further incubated with test antibody (100 μg/mL each) for 1 h at room temperature. After three washes with PBS-T, wells were incubated for 1 h at room temperature with horseradish peroxidase (HRP)-conjugated antibody against rat IgG (Jackson ImmunoResearch, Westgrove, PA, USA). After washes, bound antibodies were detected with tetramethylbenzidine substrate by measurement of absorbance at 450 nm with the subtraction of absorbance at 570 nm.

### Immunohistochemistry

Frozen sections of cartilage from an osteoarthritis patient were purchased from OriGene Technologies (Rockville, MD, USA). The sections were blocked with 2% FBS in PBS overnight at room temperature. The sections were incubated with primary antibodies for 3 h at room temperature. Either 50 μg/mL of rat α-TNF or equimolar PlGF-2_123-144_-α-TNF, 5 μg/mL of rabbit anti-human collagen II antibody (Abcam), or 25 μg/mL of mouse anti-human decorin antibody (R&D Systems, Minneapolis, MN, USA) were used as primary antibodies. After incubating with the fluorescently tagged secondary antibodies, slides were covered with ProLong Gold Antifade Mountant with DAPI (Thermo Fisher Scientific). The images were scanned with a Pannoramic digital slide scanner (3DHistech, Budapest, Hungary) and analyzed using a Pannoramic Viewer software (3DHistech).

### Cells

The murine macrophage-like cell line RAW264.7, an osteoclast precursor, was obtained from the American Type Culture Collection (ATCC; Rockville, MD, USA). Cells were cultured in Dulbecco’s modified Eagle’s medium (DMEM; Invitrogen, Carlsbad, CA, USA) supplemented with 10% FBS (Invitrogen), 100 U/mL of penicillin (Biological Industries, Cromwell, CT, USA), and 100 μg/mL of streptomycin (Biological Industries) at 37 °C in a humidified 5% CO_2_ atmosphere.

### Osteoclast differentiation from RAW264.7 cells

RAW264.7 cells were seeded on 60-mm dishes at a density of 3 × 10^6^ cells per well and cultured for 7 days in the presence of 50 ng/mL of recombinant mouse TNFα (Insight Biotechnology, Middlesex, UK), 10 ng/mL of recombinant mouse receptor activator of nuclear factor κΒ ligand (RANKL; PeproTech), and 50 to 1000 ng/mL of test antibody. The cells were then washed with PBS and stained using Acid Phosphatase, Leukocyte (TRAP) kit (MilliporeSigma) according to the manufacture’s instruction. TRAP-positive multinucleated cells having three or more nuclei were considered as osteoclasts, and their number was counted in each well. Percent inhibition was calculated from a ratio of the number of osteoclasts in each treatment to that in the untreated control. The 50% inhibitory concentration (IC_50_) values were obtained by nonlinear regression analysis (GraphPad Prism Software, LaJolla, CA, USA).

### Mice

Balb/c and athymic nude mice at 8 to 9 weeks of age were obtained from the Jackson Laboratory (Bar Harbor, ME, USA). Experiments were performed with approval from the Institutional Animal Care and Use Committee of the University of Chicago (Chicago, IL, USA).

### Collagen antibody-induced arthritis model

Arthritis was induced in Balb/c mice by intraperitoneal injection of anti-collagen antibody cocktail at 1.5 mg (Chondrex, Redmond, Washington, USA) on day − 3, followed by intraperitoneal injection of LPS at 50 μg (Chondrex) on day 0. On the day of LPS injection, mice were injected at the left hind footpad with PBS, 15 μg of PlGF-2_123-144_ peptide (theoretical equivalent amount of peptides within 100 μg of PlGF-2_123-144_-α-TNF), 100 μg of control IgG (rat IgG1 isotype control, Bio X Cell), 100 μg of unmodified α-TNF, or 0.1 or 1 μg of PlGF-2_123-144_-α-TNF. Joint swelling in the left and right hind paws was scored daily as described elsewhere [26]. The left hind paws of the collagen antibody-induced arthritis (CAIA) mice were fixed in 10% neutral formalin (MilliporeSigma), decalcified in Decalcifer II (Leica Microsystems Inc., Buffalo Grove, IL, USA), and then embedded in paraffin. The paraffin-embedded paws were sliced at 5 μm thickness and stained with hematoxylin and eosin (H&E). The images were scanned with a Pannoramic digital slide scanner and analyzed using a Pannoramic Viewer software.

### In vivo bio-distribution study

α-TNF was fluorescently labeled using sulfo-Cyanine 7 (Cy7) NHS ester (Lumiprobe, Broward, FL, USA) according to the manufacture’s instruction. PlGF-2_123-144_ peptide that was labeled with Cy7 in its N-terminus was chemically synthesized with > 90% purity by Thermo Fisher Scientific. Cy7-labeled PlGF-2_123-144_ was conjugated to α-TNF as described above. The Cy7 labeled antibody at a dose of 20 μg was injected at the left hind footpad of CAIA mice or athymic nude mice. The fluorescence level was measured using the Xenogen IVIS Imaging System 100 (Xenogen, Alameda, CA, USA) under the following conditions: f/stop: 2; optical filter excitation 745 nm; excitation 800 nm; exposure time: 5 s; small binning. Retention rates of the injected antibody immediately after the injection and that at 168 h after the injection were defined as 100% and 0%, respectively.

### Measurement of plasma concentration of the injected antibodies

CAIA mice received 1 μg of PlGF-2_123-144_-α-TNF or 100 μg of unmodified α-TNF. Blood was collected from the tail vein with a heparinized capillary at 24, 48, 72, and 96 h after the injection, and plasma was obtained by centrifugation. The injected antibodies in plasma were measured by ELISA as described above.

### Statistical analysis

Statistical analyses were performed using GraphPad Prism software, and *P* < 0.05 was considered statistically significant. Binding activities against ECM proteins were analyzed using Student’s *t* test for comparisons between PlGF-2_123-144_-α-TNF and unmodified α-TNF. The retention effect of PlGF-2_123-144_-α-TNF was analyzed in the area under the percent of retention at the injection site-time curve from 0 to 96 h (AUC_0-96h_) compared with unmodified α-TNF using Student’s t-test. The AUC_0-96h_ of plasma concentration was analyzed using Student’s t-test for comparisons between PlGF-2_123-144_-α-TNF and unmodified α-TNF. To compare the efficacy of PlGF-2_123-144_-α-TNF with unmodified α-TNF, the data on day 6 were analyzed using Tukey’s multiple comparison test.

## Results

### PlGF-2_123-144_ peptide is covalently conjugated to α-TNF

**Fig. 1:**
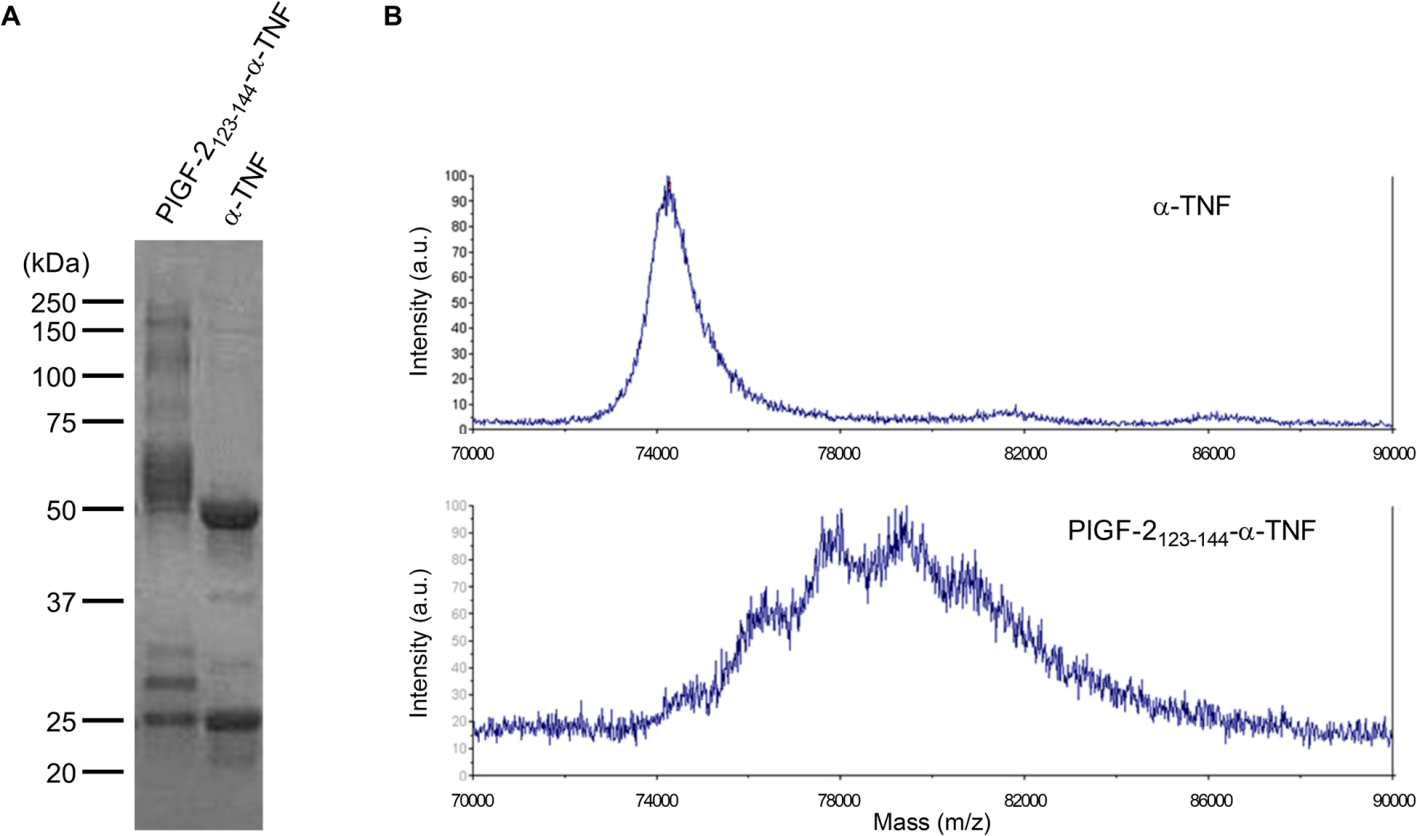
PlGF-2_123-144_ peptide conjugation with α-TNF. **a** PlGF-2_123–144_-α-TNF and unmodified α-TNF were analyzed by SDS-PAGE under reducing conditions with Coomassie blue staining. **b** Unmodified α-TNF and PlGF-2_123–144_-α-TNF were analyzed by MALDI-TOF MS. Abscissa is mass-to-charge ratio (m/z) and the ordinate is intensity of doubly charged ions

The PlGF-2_123-144_ peptide was covalently conjugated with α-TNF using a crosslinker. SDS-PAGE revealed that the molecular weights of both the light and heavy chains of α-TNF were increased (Fig. 1a). Under the stoichiometric conditions used, the α-TNF bound approximately 4.2 PlGF-2_123-144_ peptides per antibody (average 13.4 kDa shift) as measured by MALDI-TOF MS (Fig. 1b). We have previously reported that multiple PlGF-2_123-144_ peptides are conjugated to an IgG under the same reaction conditions [24], suggesting that this reaction is unaffected by antibody clones.

### PlGF-2_123-144_-α-TNF binds to multiple ECM proteins with high affinity

**Fig. 2:**
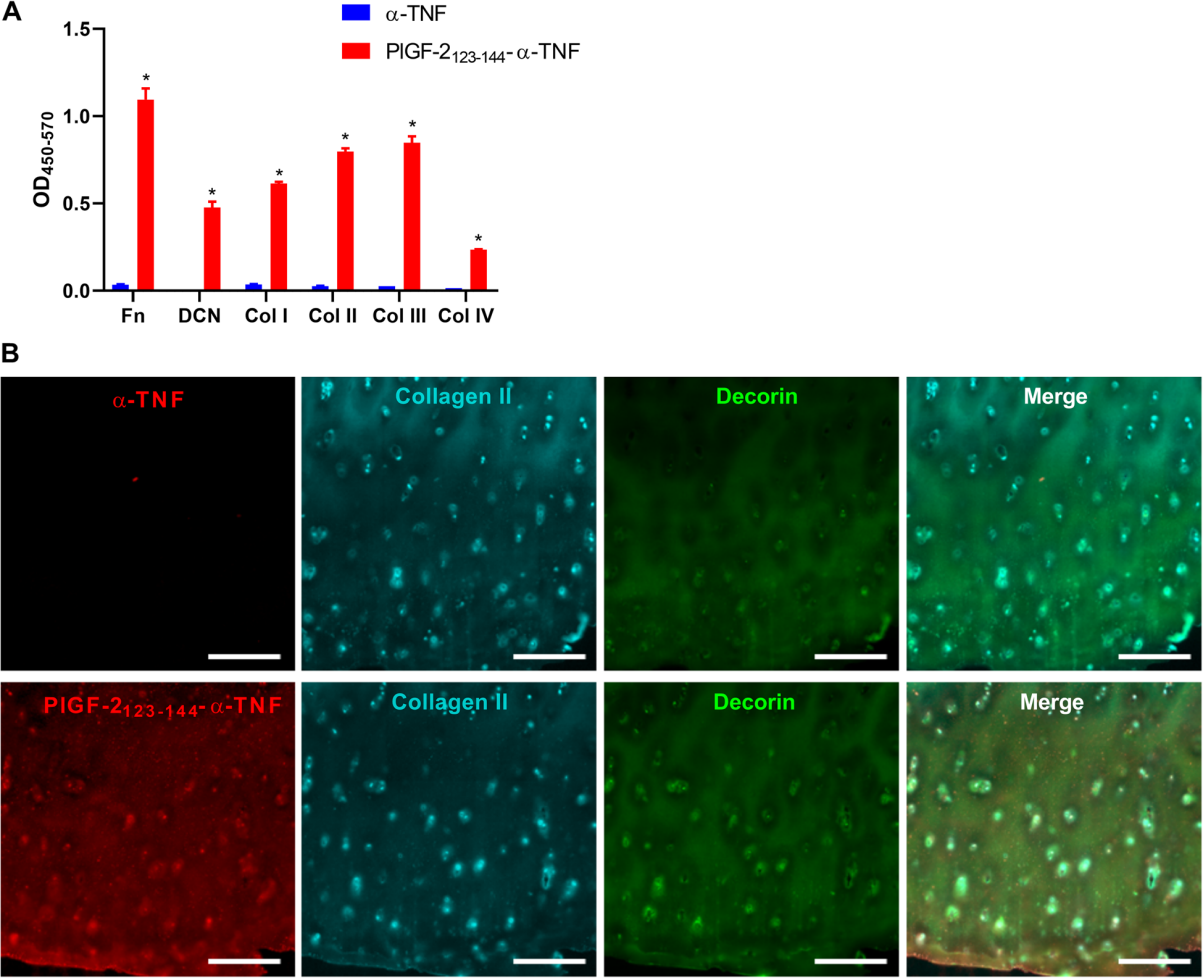
Binding of PlGF-2_123-144_-α-TNF to human cartilage. Unmodified α-TNF and PlGF-2_123-144_-α-TNF binding to fibronectin (Fn), decorin (DCN), collagen I (Col I), Col II, Col III, and Col IV were analyzed by ELISA (*n* = 3, mean + SE). *P < 0.05, compared with binding of unmodified α-TNF (Student’s *t* test). **b** Representative images of human osteoarthritis specimen probed with either unmodified α-TNF and PlGF-2_123-144_-α-TNF (red), anti-type II collagen antibody (blue), and anti-decorin antibody (green). Scale bar, 500 μm

The effect of PlGF-2_123-144_ peptide conjugation on the binding activity of α-TNF against ECM proteins was tested by ELISA. PlGF-2_123-144_-α-TNF was shown to bind to all tested ECM proteins, namely fibronectin, decorin, collagen I, collagen II, collagen III, and collagen IV; whereas no binding signal of unmodified α-TNF to these ECM proteins was detectable (Fig. 2A). In addition, PlGF-2_123-144_-α-TNF bound to ECM proteins in human cartilage specimens from an OA patient. The specimen was probed with either unmodified α-TNF or PlGF-2_123-144_-α-TNF, together with antibodies against cartilage components, namely collagen II and decorin. PlGF-2_123-144_-α-TNF bound to the regions where collagen II and decorin are rich, whereas binding of unmodified α-TNF was not detected (Fig. 2b). These data indicate that PlGF-2_123-144_-conjugation provided α-TNF with affinity against ECM proteins in cartilage.

### PlGF-2_123-144_ conjugation to α-TNF did not impair antibody neutralizing ability

**Fig. 3:**
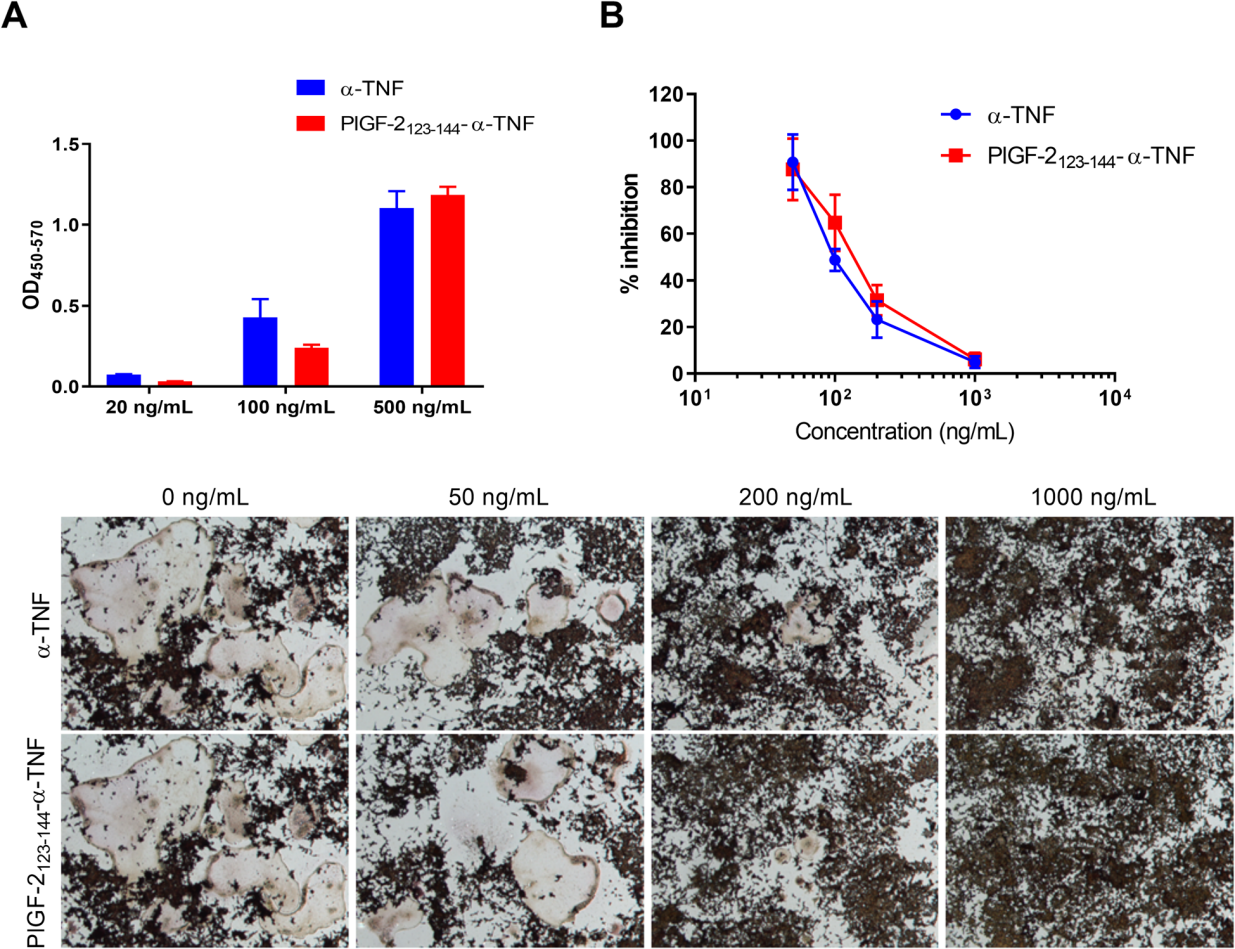
Effect of PlGF-2_123-144_ conjugation on the recognition of α-TNF against the target antigen. **a** Blocking affinities of unmodified α-TNF and PlGF-2_123-144_-α-TNF to TNFα were analyzed by ELISA (*n* = 3, mean + SE). **b** Osteoclast precursor RAW264.7 cells were incubated with TNFα and either unmodified α-TNF or PlGF-2_123-144_-α-TNF for 7 days, and then differentiated osteoclasts were stained by TRAP kit. Percent inhibition of osteoclast differentiation calculated from the ratio of the number of osteoclasts in each treatment to that in the untreated control (*n* = 6, mean ± SE). **c** Representative images of osteoclast differentiation study taken at 5-fold magnification

We tested the effect of the PlGF-2_123-144_ conjugation on antigen recognition by α-TNF. PlGF-2_123-144_-α-TNF bound to TNFα similarly to the unmodified antibody as determined by ELISA (Fig. 3a). The values of the slope (95% confidence interval [CI]) of the regression lines were 0.74 (0.57–0.90) for unmodified α-TNF and 0.82 (0.55–1.10) for PlGF-2_123-144_-α-TNF. We further investigated the activity of PlGF-2_123-144_-α-TNF and unmodified α-TNF in vitro using an assay of osteoclast differentiation, which are induced in response to TNFα from the osteoclast precursor RAW264.7 cells [27]. RAW264.7 cells were incubated with TNFα and either unmodified α-TNF or PlGF-2_123-144_-α-TNF for 7 days, and then the number of differentiated osteoclasts was determined. Osteoclast differentiation was suppressed by both antibodies in a similar manner (Fig. 3b, c). The IC_50_ values (95% CI) of unmodified α-TNF and PlGF-2_123-144_-α-TNF were 98 (87–113) ng/mL and 128 (108–155) ng/mL, respectively. These data indicate that PlGF-2_123-144_ conjugation provided α-TNF with affinity against ECM proteins, with limited impairment of its function.

### PlGF-2_123-144_-α-TNF retention is prolonged at the injected paw

**Fig. 4:**
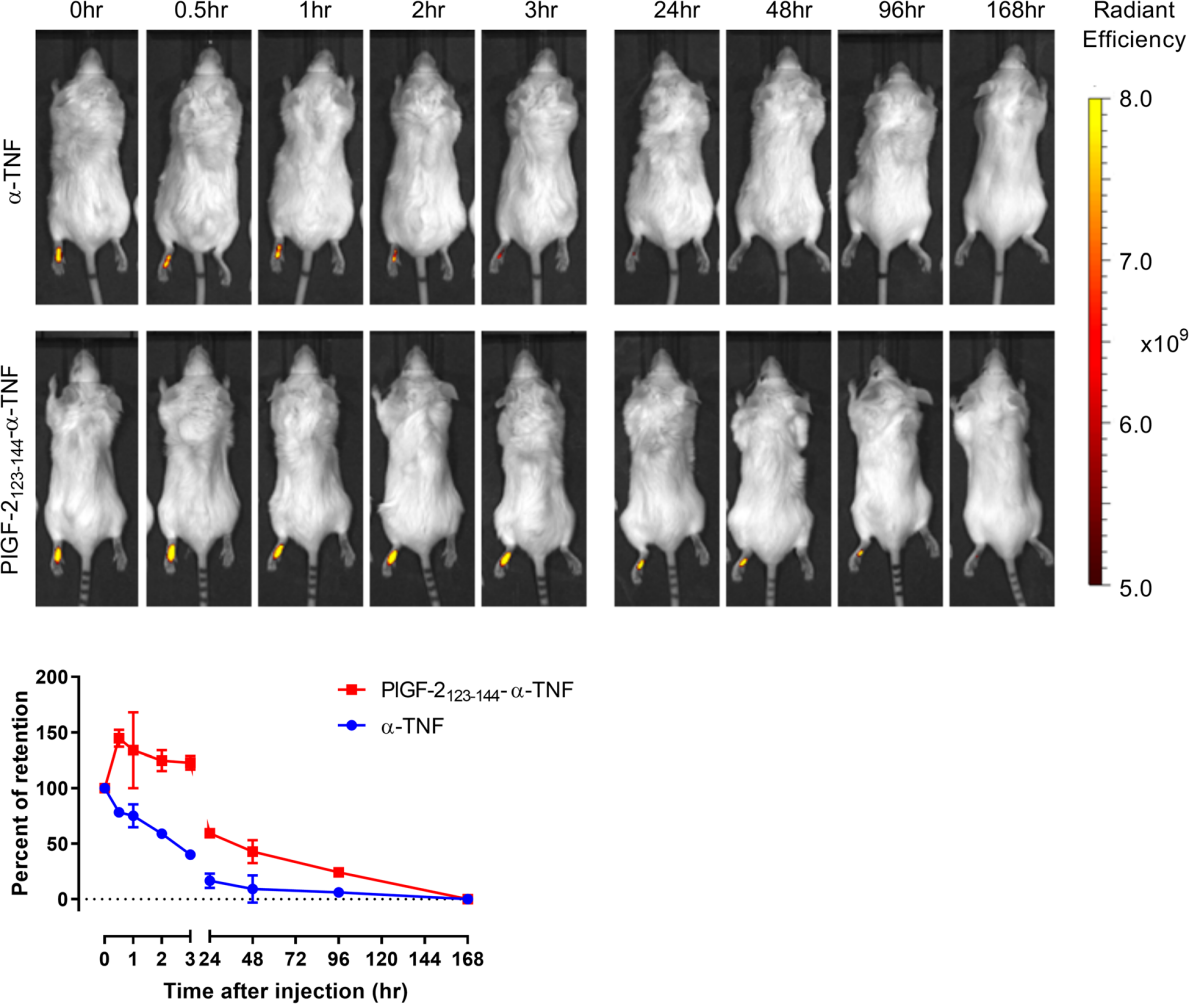
Retention of PlGF-2_123-144_-α-TNF at the injected paw. Cy7 labeled α-TNF or Cy7 labeled PlGF-2_123-144_-α-TNF was subcutaneously injected at the left hind footpad in the CAIA model. Top: Representative images of mice at 0.5, 1, 2, 3, 24, 48, 96, and 168 h after the injection. Bottom: changes in percent retention of the injected antibody at the left hind paw (*n* = 2, mean ± SE)

Retention of PlGF-2_123-144_-α-TNF at the injection site was determined by in vivo bio-distribution analysis in CAIA mice. Fluorescently labeled α-TNF or PlGF-2_123-144_-α-TNF was injected at the left hind footpad of CAIA mice on the day following arthritis induction by LPS. Whole-body fluorescence was measured immediately after the injection, and 0.5, 1, 2, 3, 24, 48, 96, and 168 h after the injection (Fig. 4). The fluorescence level was detected at the injected paw, and retention of α-TNF at the injected paw was reduced immediately after the injection. To the contrary, the fluorescence of PlGF-2_123-144_-α-TNF was maintained at a higher level at the injected paw and was detectable until 96 h after the injection. We also investigated the bio-distribution using athymic nude mice. The fluorescence of PlGF-2_123-144_-α-TNF was detected only at the injected paw until 96 h after the injection and the retention of PlGF-2_123-144_-α-TNF was significantly greater than unmodified α-TNF (Additional file 1: Figure S1). These data suggest that PlGF-2_123-144_-α-TNF binds to ECM proteins and is retained at the injection site.

### Locally injected PlGF-2_123-144_-α-TNF suppressed RA development almost completely in the CAIA model

**Fig. 5:**
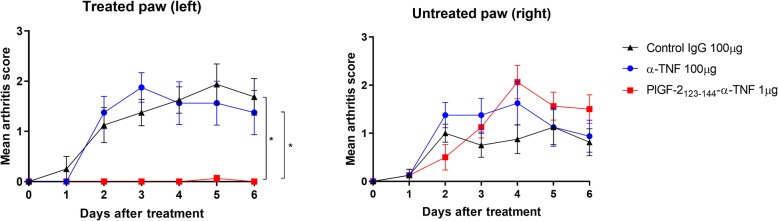
Effect of PlGF-2_123-144_-α-TNF locally injected around joints of the CAIA model. Arthritis was induced by intraperitoneal injection of anti-collagen antibodies, followed by intraperitoneal injection of LPS. On the day of LPS injection, isotype control IgG (100 μg), unmodified α-TNF (100 μg), or PlGF-2_123-144_-α-TNF (1 μg) was subcutaneously injected into the left hind paw of the CAIA mice. Arthritis scores of the treated paw and the untreated paw represent the mean ± SE from 8 mice. *P < 0.05, compared with the scores on day 6 of each group (Tukey’s multiple comparison test)

**Fig. 6:**
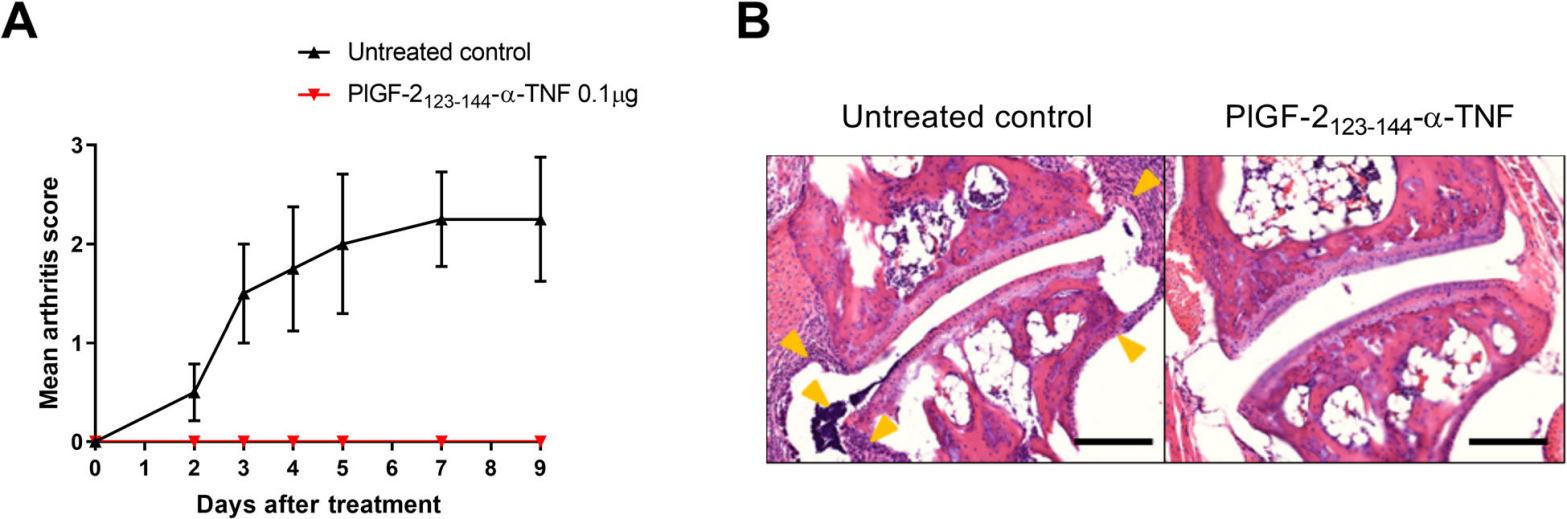
Effect of PlGF-2_123-144_-α-TNF in joint destruction of the arthritis model. Arthritis was induced by intraperitoneal injection of anti-collagen antibodies, followed by intraperitoneal injection of LPS. On the day of LPS injection, PlGF-2_123-144_-α-TNF (0.1 μg) was subcutaneously injected into the left hind paw of the CAIA mice. **a** Arthritis scores of the treated paw represent the mean ± SE from 4 mice. **b** Representative H&E image of joints on day 9 in the untreated control (left) and PlGF-2_123-144_-α-TNF-treated group (right). Arrows indicate cellular infiltrations. Scale bars, 200 μm

To examine the therapeutic efficacy of PlGF-2_123-144_-α-TNF, control IgG (100 μg), unmodified α-TNF (100 μg), or PlGF-2_123-144_-α-TNF (1 μg) was injected at the left hind footpad of CAIA mice (Fig. 5). The arthritis score was increased in both the right and left hind paws in control IgG-treated mice. Unmodified α-TNF did not suppress the score in this treatment regimen. PlGF-2_123-144_-α-TNF; however, suppressed arthritis development almost completely in the treated paw even at a 100× lower dose than unmodified α-TNF. PlGF-2_123-144_-α-TNF did not suppress arthritis development in the untreated paw, indicating its localized efficacy. We also confirmed that PlGF-2_123-144_ peptide in itself did not suppress arthritis development (Additional file 1: Figure S2), and that in contrast to unmodified α-TNF, PlGF-2_123-144_-α-TNF was not detected in plasma (Additional file 1: Figure S3). Moreover, we examined the efficacy of PlGF-2_123-144_-α-TNF at 10× further lower dose. PlGF-2_123-144_-α-TNF even at 0.1 μg completely suppressed the arthritis score at the treated paw (Fig. 6a), and joint histology in the treated paw appeared normal, whereas synovial proliferation, leukocyte infiltration, and cartilage degeneration were observed in untreated control joint (Fig. 6b). These data demonstrate that locally injected PlGF-2_123-144_-α-TNF induces robust improvement of RA therapy without systemic exposure.

## Discussion

Interest in clinical translation of intra-articularly administered therapies is growing rapidly [28]. Systemic administration of bio-therapeutics can result in only a fraction of the drug reaching the targeted tissues, with the majority of the drug being distributed to tissues irrelevant to the drug’s site of action. Localized therapy may allow for greater accumulation at target sites, better efficacy, and improved safety. Therefore, local injection is a reasonable approach for patients with monoarthritis or oligoarthritis who do not necessarily need to be exposed to systemic α-TNF treatment. In many cases, polyarthritic disorders initially present as a monoarthritis, which is potentially locally treatable. In this study, we demonstrate that PlGF-2_123-144_ peptide conjugation enhanced the local efficacy of α-TNF by at least 1000× based on dose-comparison and that the therapeutic efficacy was robust. This demonstrates that PlGF-2_123-144_ peptide conjugation enables α-TNF to be retained at joints to block TNFα signaling thoroughly and enhance its potency. Significant reduction of the effective dose and corresponding systemic exposure of locally-administered PlGF-2_123-144_-α-TNF suggests that this technology may help avoid unnecessary exposure of α-TNF to the whole body, with a corresponding reduction in adverse events.

PlGF-2_123-144_-α-TNF showed high efficacy when injected at the paw. Since the mouse paw contains many joints and disease scoring in a single joint over time is difficult, in this study we injected drugs nearby the joint space. PlGF-2_123-144_-α-TNF binds strongly to ECM proteins including collagen II, i.e., major components of cartilage and synovium, and was retained at the injection site. Therefore, these data support the concept that that PlGF-2_123-144_-α-TNF could bind to ECM in cartilage and the synovium before draining from lymphatic vessels even when PlGF-2_123-144_-α-TNF is injected intra-articularly into the joint, although further evaluation of intra-articular injection using larger animals is warranted.

Various technologies have been proposed to achieve prolonged retention of locally injected drugs, such as particle- or liposome-based drug delivery systems and modification by polypeptide conjugation [28]. Although these technologies prolong the intra-articular residence time of biologic drugs [29–31], these approaches only slightly reduced systemic circulation and there is no report to show clearly their enhanced therapeutic efficacy against disease symptoms. Therefore, our data suggest a novel approach of tissue retention of α-TNF to efficiently and locally treat RA and reduce systemic exposure due to dose reduction.

For clinical translation of ECM-binding anti-inflammatory bio-therapeutics, an advantage also lies in the use of the PlGF-2_123-144_ peptide, which naturally exists in the human body as a domain on PlGF, limiting the possibility of recognition by the immune system. It was confirmed that the PlGF-2_123-144_ peptide-conjugated antibody was capable of binding with human ECM proteins and human specimens. Also, the PlGF-2_123-144_ peptide can be conjugated to antibodies with a simple chemical reaction. The advantage of this feature is in the simplicity in production, in that it is possible to work with antibodies for which production has already been optimized. The PlGF-2_123-144_ peptide conjugation synthesis reaction for antibodies can be accomplished using chemistry that is analogous to PEGylation of proteins. Indeed, the same chemical reaction is used in antibody-drug conjugates, such as in the production of trastuzumab emtansine. These features may facilitate the development of ECM binding-drug therapy to overcome the barriers to clinical translation.

## Conclusions

We have found that installing an ECM-binding affinity to α-TNF enables it to be retained at the injection site. Moreover, ECM-binding α-TNF showed robust therapeutic efficacy in a mouse model of RA at a very low dose. This simple approach of generating an engineered ECM-binding α-TNF bio-therapeutic may hold potential for clinical translation as a local anti-inflammatory therapeutic in RA.

## Supplementary information

**Additional file 1:** **Figure S1**. Retention of PlGF-2_123-144_-α-TNF at the injected paw of athymic nude mice. Figure S2. Effect of PlGF-2_123-144_ peptide locally injected around joints of the CAIA model. Figure S3. Changes in plasma concentrations after local injection to CAIA mice.

## Data Availability

All data generated or analyzed during this study are included in this article and its supplementary information files.

## Abbreviations

AUC: Area under the curve
CAIA: Collagen antibody-induced arthritis model
Cy7: Cyanine 7
ECM: Extracellular matrix
GF: Growth factors
HBD: Heparin-binding domain
HRP: Horseradish peroxidase
LPS: Lipopolysaccharides
MALDI-TOF MS: Matrix-assisted laser desorption/ionization–time-of-flight mass spectrometry
PBS: Phosphate-buffered saline
PBS-T: 2% BSA in PBS with 0.05% Tween 20
PlGF-2_123-144_: HBD of placenta growth factor-2
PlGF-2_123-144_-α-TNF: PlGF-2_123-144_-conjugated α-TNF
RA: Rheumatoid arthritis
SDS-PAGE: Sodium dodecyl sulfate-polyacrylamide gel electrophoresis
α-TNF: Anti-tumor necrosis factor-α antibody

## References

[1] Firestein GS (2003). Evolving concepts of rheumatoid arthritis. Nature.

[2] McInnes IB, Schett G (2011). The pathogenesis of rheumatoid arthritis. N Engl J Med.

[3] Moreland LW, Schiff MH, Baumgartner SW, Tindall EA, Fleischmann RM, Bulpitt KJ, Weaver AL, Keystone EC, Furst DE, Mease PJ (1999). Etanercept therapy in rheumatoid arthritis. A randomized, controlled trial. Ann Intern Med.

[4] Maini R, St Clair EW, Breedveld F, Furst D, Kalden J, Weisman M, Smolen J, Emery P, Harriman G, Feldmann M (1999). Infliximab (chimeric anti-tumour necrosis factor alpha monoclonal antibody) versus placebo in rheumatoid arthritis patients receiving concomitant methotrexate: a randomised phase III trial. ATTRACT Study Group. Lancet.

[5] Weinblatt ME, Keystone EC, Furst DE, Moreland LW, Weisman MH, Birbara CA, Teoh LA, Fischkoff SA, Chartash EK (2003). Adalimumab, a fully human anti-tumor necrosis factor alpha monoclonal antibody, for the treatment of rheumatoid arthritis in patients taking concomitant methotrexate: the ARMADA trial. Arthritis Rheum.

[6] Nishimoto N, Yoshizaki K, Miyasaka N, Yamamoto K, Kawai S, Takeuchi T, Hashimoto J, Azuma J, Kishimoto T (2004). Treatment of rheumatoid arthritis with humanized anti-interleukin-6 receptor antibody: a multicenter, double-blind, placebo-controlled trial. Arthritis Rheum.

[7] Bongartz T, Sutton AJ, Sweeting MJ, Buchan I, Matteson EL, Montori V (2006). Anti-TNF antibody therapy in rheumatoid arthritis and the risk of serious infections and malignancies: systematic review and meta-analysis of rare harmful effects in randomized controlled trials. JAMA.

[8] Dixon WG, Hyrich KL, Watson KD, Lunt M, Galloway J, Ustianowski A, Consortium BSRBRCC, Symmons DP, Register BSRB (2010). Drug-specific risk of tuberculosis in patients with rheumatoid arthritis treated with anti-TNF therapy: results from the British Society for Rheumatology Biologics Register (BSRBR). Ann Rheum Dis.

[9] Bellis E, Scire CA, Carrara G, Adinolfi A, Batticciotto A, Bortoluzzi A, Cagnotto G, Caprioli M, Canzoni M, Cavatorta FP (2016). Ultrasound-detected tenosynovitis independently associates with patient-reported flare in patients with rheumatoid arthritis in clinical remission: results from the observational study STARTER of the Italian Society for Rheumatology. Rheumatology (Oxford).

[10] Lisbona MP, Solano A, Ares J, Almirall M, Salman-Monte TC, Maymo J (2016). ACR/EULAR definitions of remission are associated with lower residual inflammatory activity compared with DAS28 remission on hand MRI in rheumatoid arthritis. J Rheumatol.

[11] Vreju FA, Filippucci E, Gutierrez M, Di Geso L, Ciapetti A, Ciurea ME, Salaffi F, Grassi W (2016). Subclinical ultrasound synovitis in a particular joint is associated with ultrasound evidence of bone erosions in that same joint in rheumatoid patients in clinical remission. Clin Exp Rheumatol.

[12] Mjaavatten MD, Haugen AJ, Helgetveit K, Nygaard H, Sidenvall G, Uhlig T, Kvien TK (2009). Pattern of joint involvement and other disease characteristics in 634 patients with arthritis of less than 16 weeks' duration. J Rheumatol.

[13] Jones G, Nash P, Hall S (2017). Advances in rheumatoid arthritis. Med J Aust.

[14] Bajpayee AG, Grodzinsky AJ (2017). Cartilage-targeting drug delivery: can electrostatic interactions help?. Nat Rev Rheumatol.

[15] Wallis WJ, Simkin PA, Nelp WB (1987). Protein traffic in human synovial effusions. Arthritis Rheum.

[16] Bliddal H, Terslev L, Qvistgaard E, Konig M, Holm CC, Rogind H, Boesen M, Danneskiold-Samsoe B, Torp-Pedersen S (2006). A randomized, controlled study of a single intra-articular injection of etanercept or glucocorticosteroids in patients with rheumatoid arthritis. Scand J Rheumatol.

[17] Aalbers C, Gerlag D, Vos K, Vervoordeldonk M, Landewe R, Tak PP (2015). Intra-articular etanercept treatment in inflammatory arthritis: a randomized double-blind placebo-controlled proof of mechanism clinical trial validating TNF as a potential therapeutic target for local treatment. Joint Bone Spine.

[18] Martino MM, Briquez PS, Ranga A, Lutolf MP, Hubbell JA (2013). Heparin-binding domain of fibrin (ogen) binds growth factors and promotes tissue repair when incorporated within a synthetic matrix. Proc Natl Acad Sci U S A.

[19] Martino MM, Tortelli F, Mochizuki M, Traub S, Ben-David D, Kuhn GA, Muller R, Livne E, Eming SA, Hubbell JA (2011). Engineering the growth factor microenvironment with fibronectin domains to promote wound and bone tissue healing. Sci Transl Med.

[20] De Laporte L, Rice JJ, Tortelli F, Hubbell JA (2013). Tenascin C promiscuously binds growth factors via its fifth fibronectin type III-like domain. PLoS One.

[21] Tortelli F, Pisano M, Briquez PS, Martino MM, Hubbell JA (2013). Fibronectin binding modulates CXCL11 activity and facilitates wound healing. PLoS One.

[22] Maile LA, Busby WH, Sitko K, Capps BE, Sergent T, Badley-Clarke J, Ling Y, Clemmons DR (2006). The heparin binding domain of vitronectin is the region that is required to enhance insulin-like growth factor-I signaling. Mol Endocrinol.

[23] Martino MM, Briquez PS, Guc E, Tortelli F, Kilarski WW, Metzger S, Rice JJ, Kuhn GA, Muller R, Swartz MA (2014). Growth factors engineered for super-affinity to the extracellular matrix enhance tissue healing. Science.

[24] Ishihara Jun, Fukunaga Kazuto, Ishihara Ako, Larsson Hans M., Potin Lambert, Hosseinchi Peyman, Galliverti Gabriele, Swartz Melody A., Hubbell Jeffrey A. (2017). Matrix-binding checkpoint immunotherapies enhance antitumor efficacy and reduce adverse events. Science Translational Medicine.

[25] Ishihara J, Ishihara A, Potin L, Hosseinchi P, Fukunaga K, Damo M, Gajewski TF, Swartz MA, Hubbell JA (2018). Improving efficacy and safety of agonistic anti-CD40 antibody through extracellular matrix affinity. Mol Cancer Ther.

[26] Kagari T, Doi H, Shimozato T (2002). The importance of IL-1 beta and TNF-alpha, and the noninvolvement of IL-6, in the development of monoclonal antibody-induced arthritis. J Immunol.

[27] Kobayashi K, Takahashi N, Jimi E, Udagawa N, Takami M, Kotake S, Nakagawa N, Kinosaki M, Yamaguchi K, Shima N (2000). Tumor necrosis factor alpha stimulates osteoclast differentiation by a mechanism independent of the ODF/RANKL-RANK interaction. J Exp Med.

[28] Evans CH, Kraus VB, Setton LA (2014). Progress in intra-articular therapy. Nat Rev Rheumatol.

[29] Erdemli O, Ozen S, Keskin D, Usanmaz A, Batu ED, Atilla B, Tezcaner A (2014). In vitro evaluation of effects of sustained anti-TNF release from MPEG-PCL-MPEG and PCL microspheres on human rheumatoid arthritis synoviocytes. J Biomater Appl.

[30] Betre H, Liu W, Zalutsky MR, Chilkoti A, Kraus VB, Setton LA (2006). A thermally responsive biopolymer for intra-articular drug delivery. J Control Release.

[31] Kimmerling KA, Furman BD, Mangiapani DS, Moverman MA, Sinclair SM, Huebner JL, Chilkoti A, Kraus VB, Setton LA, Guilak F (2015). Sustained intra-articular delivery of IL-1RA from a thermally-responsive elastin-like polypeptide as a therapy for post-traumatic arthritis. Eur Cell Mater.

