# Supplementary material for: Conferring extracellular matrix affinity enhances local therapeutic efficacy of anti-TNF-α antibody in a murine model of rheumatoid arthritis
Source: Arthritis Res Ther. 2019 Dec 23;21:298. doi: 10.1186/s13075-019-2075-8 (PMC6929495; doi:10.1186/s13075-019-2075-8)
Supplement: Supplementary file 1 — Additional file 1: Figure S1. Retention of PlGF-2123-144-α-TNF at the injected paw of athymic nude mice. Figure S2. Effect of PlGF-2123-144 peptide locally injected around joints of the CAIA model. Figure S3. Changes in plasma concentrations after local injection to CAIA mice. [file 13075_2019_2075_MOESM1_ESM.docx]

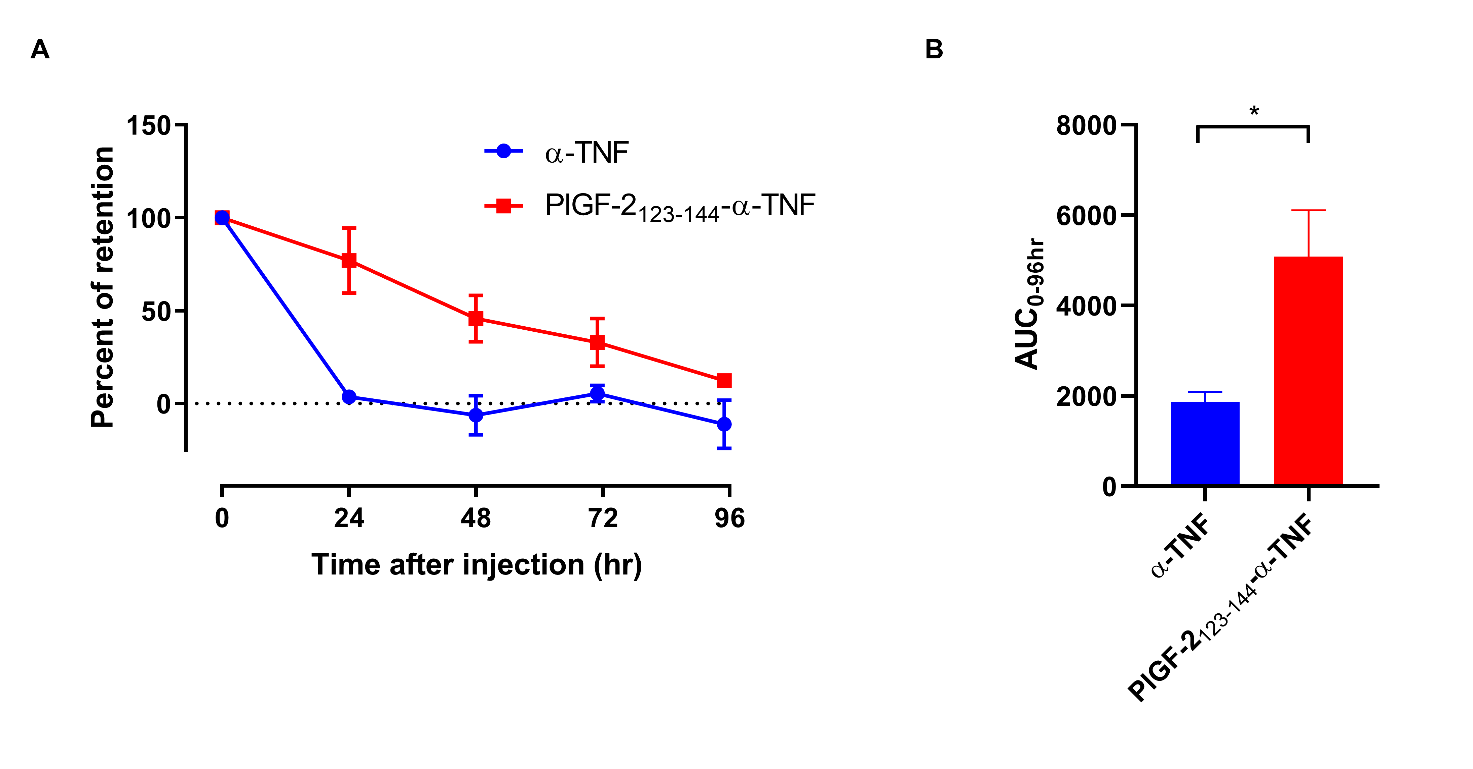


**Figure S1.** Retention of PlGF-2_123-144_-α-TNF at the injected paw of athymic nude mice. Cy7 labeled α-TNF or Cy7 labeled PlGF-2_123-144_-α-TNF was subcutaneously injected at the left hind footpad in the athymic nude mice. (A) Changes in percent retention of the injected antibody at the left hind paw (n=3, mean ± SE). (B) Area under the percent of retention-time curve from 0 to 96 hours (n=3, mean + SE). *P<0.05, compared with the AUC_0-96hr_ between α-TNF and PlGF-2_123-144_-α-TNF.


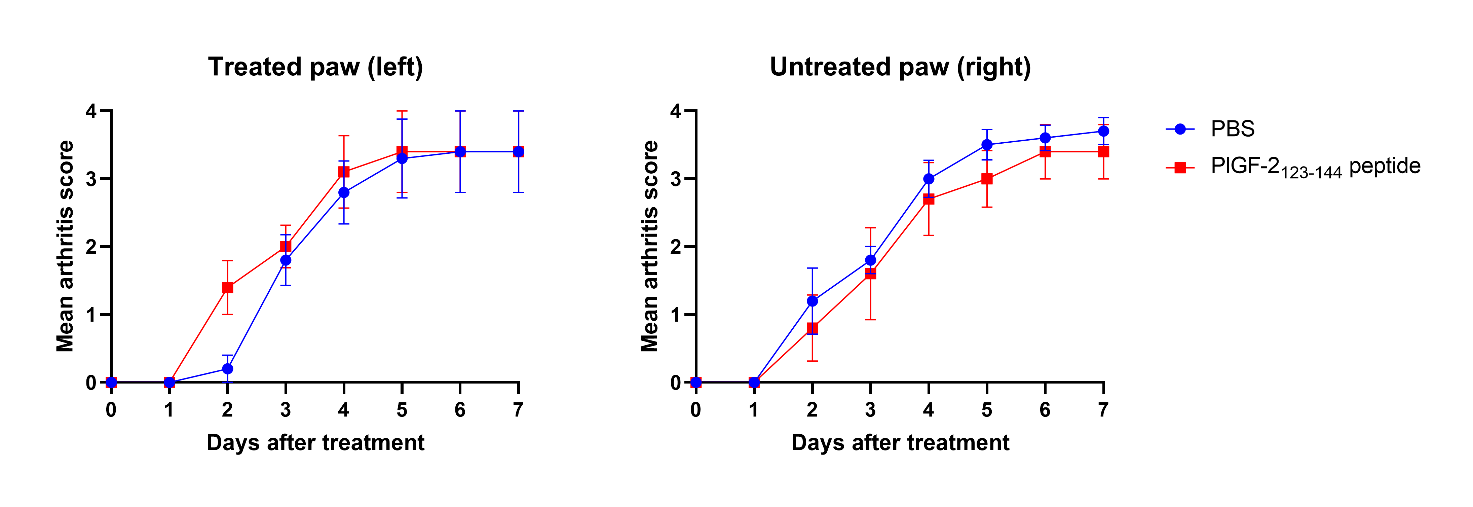


**Figure S2.** Effect of PlGF-2_123-144_ peptide locally injected around joints of the CAIA model. Arthritis was induced by intraperitoneal injection of anti-collagen antibodies, followed by intraperitoneal injection of LPS. On the day of LPS injection, PBS or 15 μg of PlGF-2_123-144_ peptide was subcutaneously injected into the left hind paw of the CAIA mice. Arthritis scores of the treated paw and the untreated paw represent the mean ± SE from 5 mice.


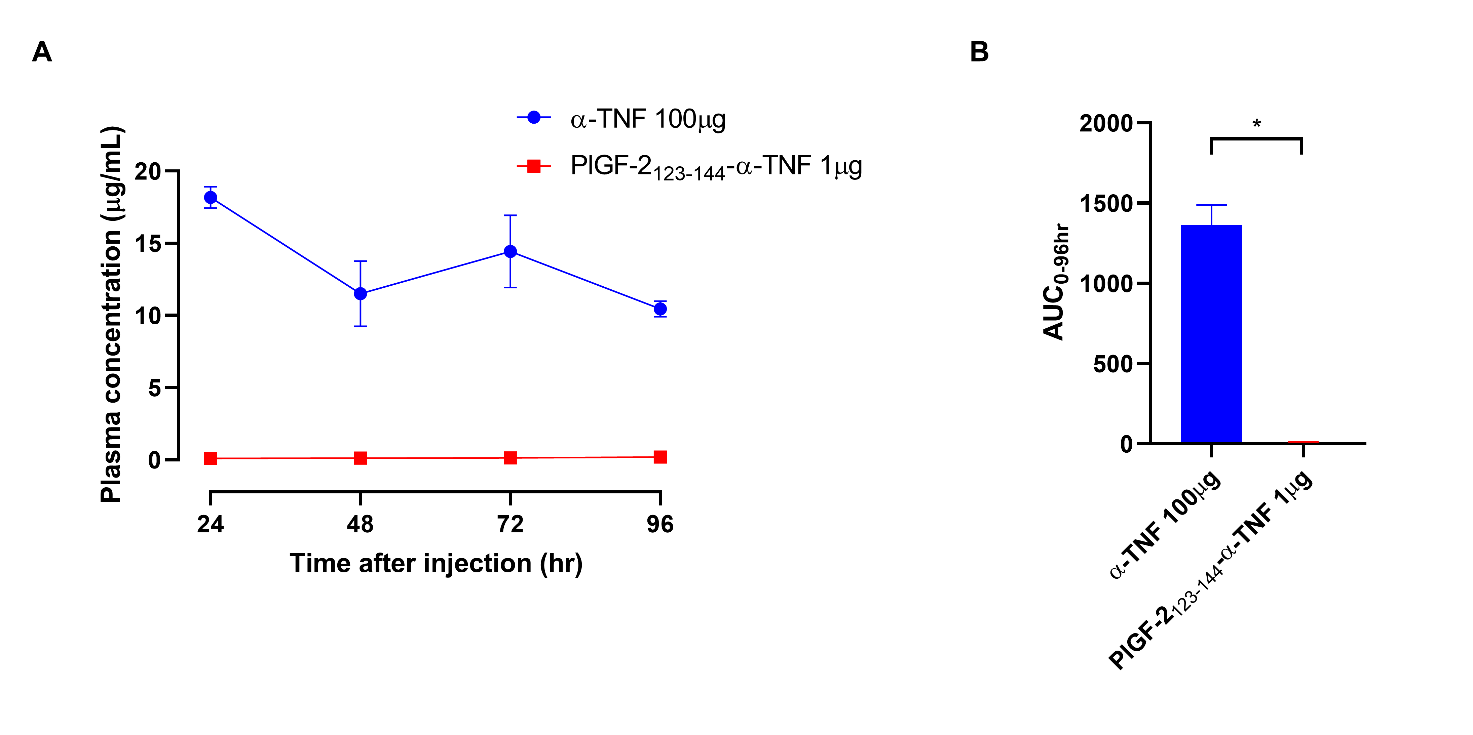


**Figure S3.** Changes in plasma concentrations after local injection in CAIA mice. Arthritis was induced by intraperitoneal injection of anti-collagen antibodies, followed by intraperitoneal injection of LPS. On the day of LPS injection, 100 μg of α-TNF or 1 μg of PlGF-2_123-144_-α-TNF was subcutaneously injected into the left hind paw of the CAIA mice. Blood was collected at 24, 48, 72, and 96 hours after the injection and the injected antibodies in plasma was measured by ELISA. (A) Data represent mean ± SE from 3 mice. (B) Area under the plasma concentration-time curve from 0 to 96 hours (n=3, mean + SE). *P<0.05, compared with the AUC_0-96hr_ between α-TNF and PlGF-2_123-144_-α-TNF.
